# Genome-wide DNA methylation reveals potential epigenetic mechanism of age-dependent viral susceptibility in grass carp

**DOI:** 10.1186/s12979-022-00285-w

**Published:** 2022-06-02

**Authors:** Libo He, Xinyu Liang, Qian Wang, Cheng Yang, Yongming Li, Lanjie Liao, Zuoyan Zhu, Yaping Wang

**Affiliations:** 1 State Key Laboratory of Freshwater Ecology and Biotechnology, Institute of Hydrobiology, Chinese Academy of Sciences, Wuhan, 430072 China; 2 University of Chinese Academy of Sciences, Beijing, 100049 China; 3 Innovative Academy of Seed Design, Chinese Academy of Sciences, Beijing, 100101 China

**Keywords:** Genome-wide DNA methylation, Grass carp, Grass carp reovirus, Age-dependent viral susceptibility, Epigenetic mechanism, Immune response, Biosynthesis, Energy metabolism

## Abstract

**Background:**

Grass carp are an important farmed fish in China that are infected by many pathogens, especially grass carp reovirus (GCRV). Notably, grass carp showed age-dependent susceptibility to GCRV; that is, grass carp not older than one year were sensitive to GCRV, while those over three years old were resistant to this virus. However, the underlying mechanism remains unclear. Herein, whole genome-wide DNA methylation and gene expression variations between susceptible five-month-old (FMO) and resistant three-year-old (TYO) grass carp were investigated aiming to uncover potential epigenetic mechanisms.

**Results:**

Colorimetric quantification revealed that the global methylation level in TYO fish was higher than that in FMO fish. Whole-genome bisulfite sequencing (WGBS) of the two groups revealed 6214 differentially methylated regions (DMRs) and 4052 differentially methylated genes (DMGs), with most DMRs and DMGs showing hypermethylation patterns in TYO fish. Correlation analysis revealed that DNA hypomethylation in promoter regions and DNA hypermethylation in gene body regions were associated with gene expression. Enrichment analysis revealed that promoter hypo-DMGs in TYO fish were significantly enriched in typical immune response pathways, whereas gene body hyper-DMGs in TYO fish were significantly enriched in terms related to RNA transcription, biosynthesis, and energy production. RNA-seq analysis of the corresponding samples indicated that most of the genes in the above terms were upregulated in TYO fish. Moreover, gene function analysis revealed that the two genes involved in energy metabolism displayed antiviral effects.

**Conclusions:**

Collectively, these results revealed genome-wide variations in DNA methylation between grass carp of different ages. DNA methylation and gene expression variations in genes involved in immune response, biosynthesis, and energy production may contribute to age-dependent susceptibility to GCRV in grass carp. Our results provide important information for disease-resistant breeding programs for grass carp and may also benefit research on age-dependent diseases in humans.

**Supplementary Information:**

The online version contains supplementary material available at 10.1186/s12979-022-00285-w.

## Background

Grass carp (*Ctenopharyngodon idellus*) are an important farmed fish in China, accounting for more than 18% of the total freshwater aquaculture production in this country. The production of grass carp reached 5.53 million tons in 2019, indicating the great commercial value of this fish [1]. Nevertheless, grass carp reovirus (GCRV)-induced hemorrhagic disease is a major threat to the grass carp aquaculture industry [2]. Consequently, the disease susceptibility of grass carp to GCRV is of major interest to fish-breeding geneticists aiming to identify strategies for disease-resistant breeding [3–7]. Our previous study revealed that age is an important factor that influences the susceptibility of grass carp to GCRV infection. GCRV infection in grass carp not older than one year caused death and hemorrhagic symptoms, while no dead individuals were observed in grass carp over three years of age after virus infection [8]. Moreover, we found that the level of immune response and host biosynthesis and metabolism are involved in the age-dependent viral susceptibility of grass carp [9]. However, the reason for the different immune response levels or host biosynthesis and metabolism levels in grass carp of different ages remains poorly understood.

In mammals, age is also a crucial factor that affects disease outcomes [10]. Pseudorabies virus (PRV) infection causes more severe clinical diseases in newborns than in older individuals [11]. Infants and young children are more sensitive to reovirus-induced encephalitis than adults [12]. Mice display an age-dependent acceleration of mortality due to influenza virus (IAV) infection, and are useful for modeling human aging and the outcomes of IAV infection [13]. It has been suggested that the maturation of the innate immune system and the expression of type I interferon genes may contribute to age-related viral susceptibility [14]; however, the reason for the differential gene expression patterns between young and adult patients is still unclear.

DNA methylation is an important mediator of gene expression regulation, and many genes have been reported to be influenced by DNA methylation at different ages [15–17]. Transporter associated with antigen processing 1 (TAP1) gene promoter methylation level was decreased, while mRNA expression was increased in Sutai piglets from birth to weaning age (8, 18, 30, and 35 days old), which may contribute to the resistance of piglets to *Escherichia coli* F18 at 35 days of age [18]. Genome-wide DNA methylation analysis of CD4+ and CD8+ T cells from younger and older individuals has revealed an increased number of methylation changes and higher methylome variation in CD8+ T cells with age, implying a link between age-related epigenetic changes and impaired T cell function [19]. A genome-wide DNA methylation survey of skeletal muscle between young and old healthy humans showed a dynamic interrelationship between DNA methylation, gene expression, age, and exercise [20]. Therefore, DNA methylation appears to play an important role in age-related gene expression and is thought to underlie many age-related physiological phenomena, such as age-dependent susceptibility to viral infection.

In this study, we investigated genome-wide DNA methylation and gene expression variations between susceptible five-month-old (FMO) grass carp and resistant three-year-old (TYO) grass carp to reveal the potential epigenetic mechanism of age-dependent viral susceptibility in grass carp. Differentially methylated regions (DMRs) and differentially expressed genes (DEGs) were identified. The correlation between DNA methylation and gene expression was determined. Moreover, the roles of the two metabolism-related genes during viral infection were investigated. We believe our results will provide valuable information for disease-resistant breeding of grass carp and may also benefit research on age-dependent diseases in humans.

## Results

### Global methylation profile and whole-genome bisulfite sequencing

**Fig. 1:**
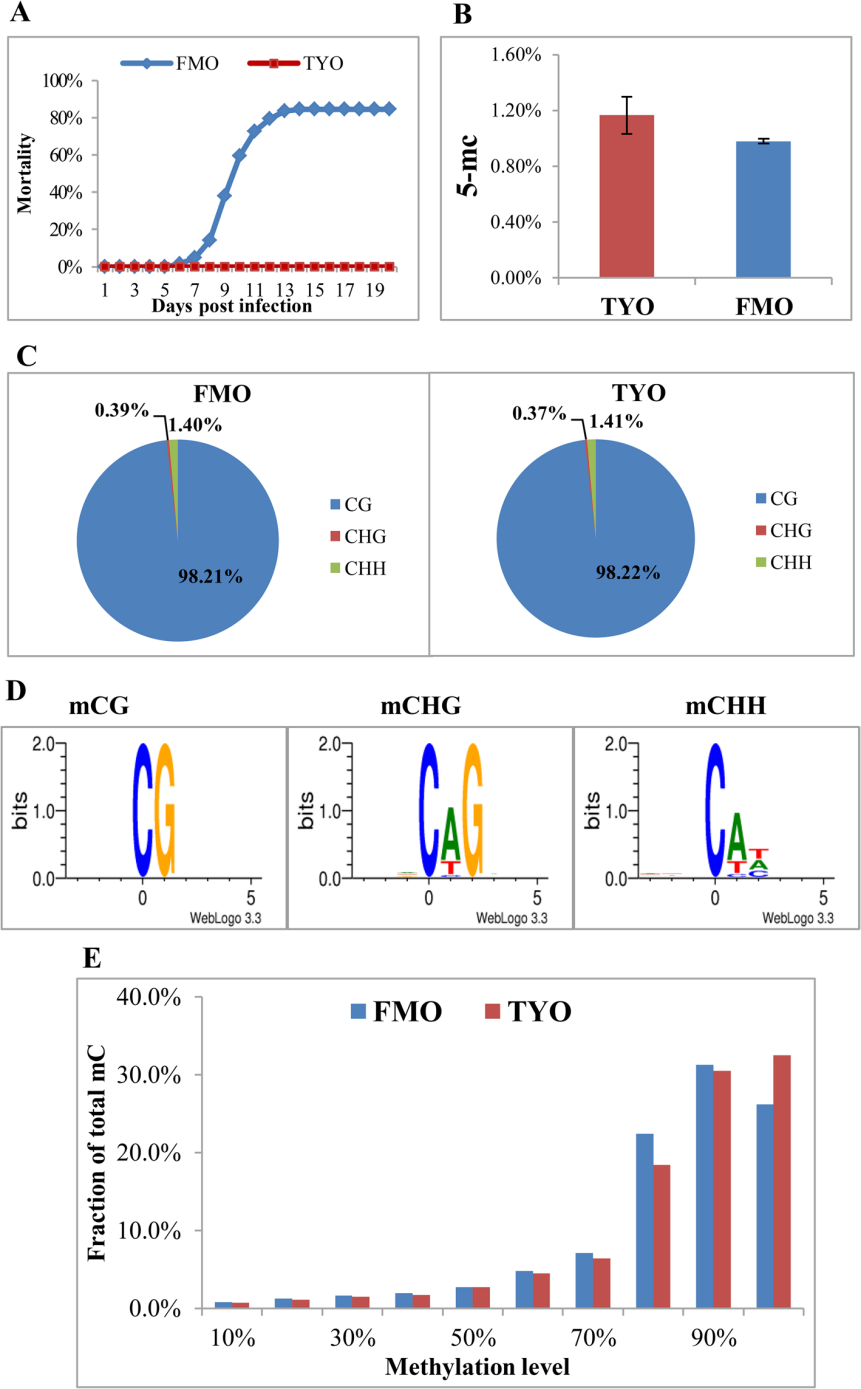
Age-dependent susceptibility to grass carp reovirus (GCRV) and global methylation profile of the two fish groups. **A** The cumulative mortality of the two grass carp groups after GCRV infection. **B** Global DNA methylation level of two grass carp groups. Spleen samples from the two groups were obtained, and the global DNA methylation level (5-mc) was analyzed. Data are shown as the mean ± standard deviation of three replicates. **C** The percentage of methylcytosines in CG, CHG, and CHH contexts. **D** Logo plots of the sequences proximal to sites of mCG, mCHG, and mCHH contexts. **E** Distribution of DNA methylation levels in the two groups. The y axis indicates the fraction of all mCs that display each methylation level (x axis), where the methylation level is the mC/C ratio at each cytosine

A viral challenge experiment was carried out for approximately 200 FMO and 200 TYO grass carps. For the FMO group, a total mortality of 85% was reached at 14 days, with the first death recorded as early as 6 days post-infection (Fig. 1A). However, none of the individuals in the TYO group died (Fig. 1A). This result was consistent with our previous report [9], further confirming age-dependent susceptibility to GCRV in grass carp. Moreover, we analyzed the global DNA methylation status of spleens from the two groups by colorimetric quantification of 5-methylcytosine. As shown in Fig. 1B, the global methylation level in TYO fish was higher than that in FMO fish, although this difference was not significant.

To further reveal the potential epigenetic mechanisms underlying age-dependent viral susceptibility in grass carp, we performed whole-genome bisulfite sequencing (WGBS) on samples collected from FMO and TYO fish at base-pair resolution. Three replicates were processed for each group, yielding six libraries (*n* = 5 for each library). All libraries were sequenced on an Illumina HiSeq X Ten platform to generate 150 bp paired-end reads. Each library yielded > 33.68 GB clean base and > 23.06 × mean coverage depth. The bisulfite conversion rates for all libraries exceeded 99.88%. Moreover, for all libraries, more than 82.78% of the genomic sites were covered by at least five unique reads, and more than 70.70% of the sites were covered by at least ten unique reads (Table 1). Pearson’s correlation analysis and principal component analysis of the six libraries showed that samples from each group clustered together (Additional file 1: Fig. S1A and S1B). Collectively, these results confirm the high quality and repeatability of the WGBS data and its suitability for further analysis. WGBS data were deposited in the Sequence Read Archive (SRA) at the National Center for Biotechnology Information (NCBI) (accession number: PRJNA638254).

**Table 1:** Summary of WGBS data

Samples	replicates	clean bases(G)	Mapped reads	Unique Mapping rate (%)	BS conversion rate (%)	Mean coverage (×)	1× coverage (%)	5× coverage (%)	10× coverage (%)
FMO	FMO-a	37.55	91,721,180	69.78	99.88	27	91.6	85.56	76.16
	FMO-b	36.75	78,836,313	60.79	99.90	23.06	91.07	82.78	70.7
	FMO-c	33.68	82,684,543	70.36	99.91	23.86	91.85	86.2	75.31
TYO	TYO-a	36.45	95,438,882	75.26	99.89	28.5	91.57	84.77	74.1
	TYO-b	38.15	90,027,308	67.42	99.88	26.83	91.63	83.57	70.99
	TYO-c	34.73	84,701,266	69.7	99.89	25.31	91.02	83.57	71.52

In the detected methylation sites, the average methylated cytosine (mC) percentages of whole genomic cytosines were 4.99 and 4.80% for FMO and TYO fish, respectively. In the CG context, approximately half of the cytosines were methylated (51.42% in FMO fish and 49.40% in TYO fish), whereas the methylation rates of cytosines in the CHG and CHH contexts (where H is A, C, or T) were no more than 0.10 and 0.09%, respectively (Additional file 3: Table S1). Among the mC sites that were identified, more than 98% were in the CG context, whereas no more than 0.4 and 1.5% were in the CHG and CHH contexts, respectively (Fig. 1C). The genome sequence preference proximal to the sites of methylated CG (mCG), methylated CHG (mCHG), and methylated CHH (mCHH) contexts was also analyzed. There was no sequence preference in the mCG-flanking regions or upstream of the mCHG and mCHH contexts; however, the base following the mCHG and mCHH contexts was almost always adenine, followed by thymine, while cytosine was observed less often (Fig. 1D). The methylation level (ML), defined as the proportion of reads covering each mC relative to the total reads covering the sites, was calculated. The results showed that more than 80% of the mC sites displayed high ML (ML ≥ 70%) (Fig. 1E).

### Gene methylation profile

**Fig. 2:**
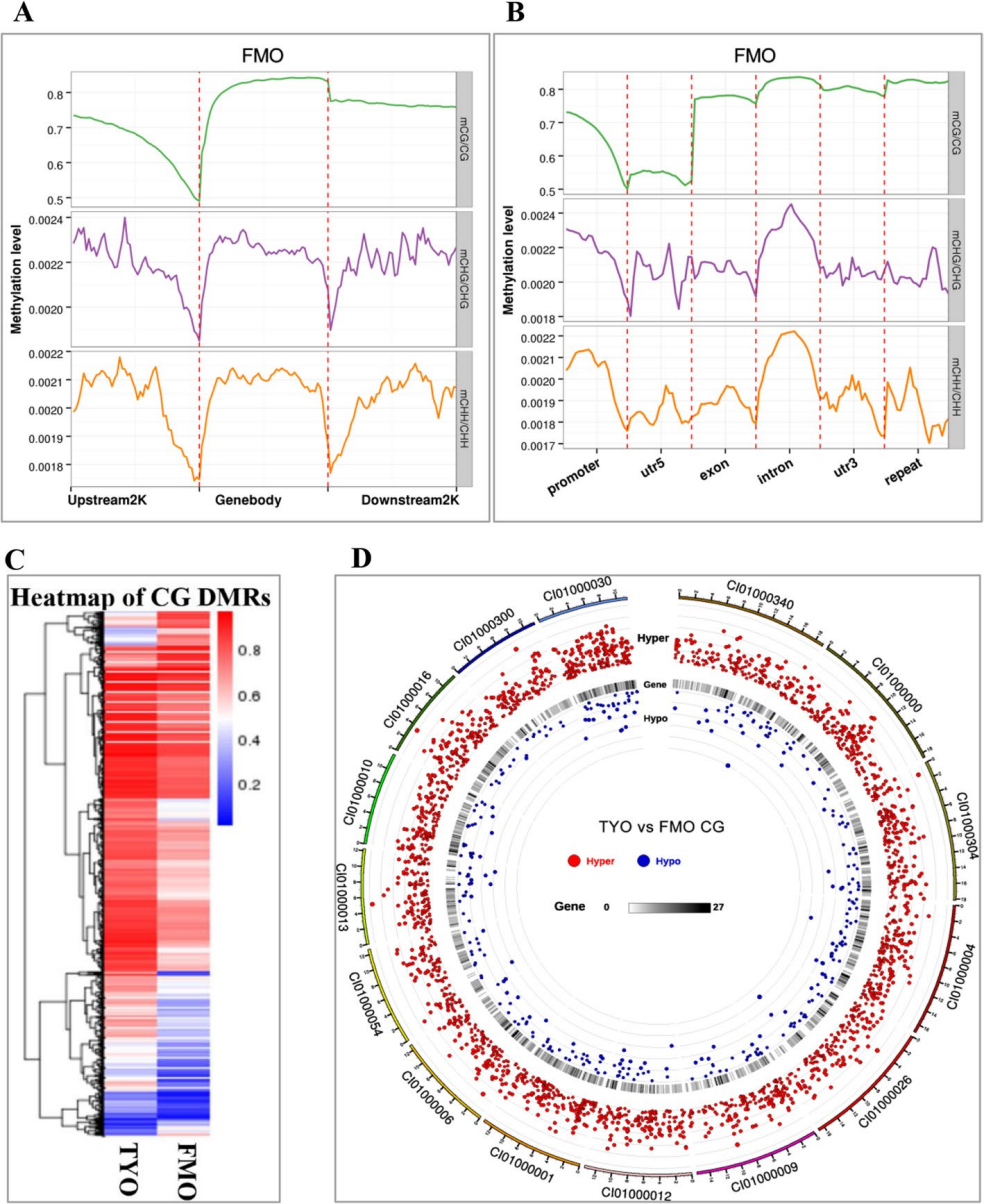
Distribution of mCs in different genome regions and the differentially methylated regions (DMRs) identified between the two groups. **A** Relative methylation level in gene body regions and of their upstream and downstream regions. **B** Relative methylation level in different gene elements (promoter, 5′ UTR, exon, intron, 3′ UTR, and repeat regions). **C** The heatmap of DMRs. Red represents hypermethylation, whereas blue represents hypomethylation. **D** The distribution of DMRs in the genome. Black dots indicate hyper-DMRs, while gray dots represent hypo-DMRs

To characterize the methylation profile of grass carp genes, the relative ML in the context of gene regions and their upstream and downstream regions was calculated. In general, the relative ML of mCGs in the gene body regions was higher than that in the 5′ upstream and 3′ downstream regions in both groups, whereas the ML in mCHG and mCHH was more complex (Fig. 2A and Additional file 1: Fig. S1C). Interestingly, a sharp decrease in ML was observed across the boundaries of gene body regions and upstream or downstream regions (Fig. 2A). We further divided the genome into different elements, including the promoter, 5′ untranslated region (UTR), exon, intron, 3′ UTR, and repeats. The results suggested that the promoter and 5′ UTR had relatively low ML in mCG, followed by the exon, whereas the repeats had the highest ML (Fig. 2B and Additional file 1: Fig. S1D).

### DMRs between FMO and TYO fish

To further investigate the epigenetic differences between the two fish groups, the WGBS data of TYO fish were compared with those of FMO fish, and DMRs were identified using DSS software. As most mC sites occurred in CG contexts (Fig. 1B), only DMRs in CG contexts were considered for further study. A total of 6214 DMRs were identified between the two fish groups, including 5261 hyper-DMRs (hypermethylation in TYO fish compared with FMO fish) and 953 hypo-DMRs (hypomethylation in TYO fish compared with FMO fish) (Additional file 4: Table S2). The number of hyper-DMRs was greater than that of hypo-DMRs. Interestingly, we also found that the mean ML of DMRs in TYO fish was higher than that in FMO fish (Additional File 1: Fig. S1E). The heatmap of the DMRs also showed a similar result (Fig. 2C). The length distribution of the DMRs was analyzed, and the results showed that most of them were shorter than 400 bp (Additional file 1: Fig. S1F). Circos analysis in a circular layout revealed that the DMRs were distributed uniformly in the genome, except for supercontig C101000030 (Fig. 2D), in which more DMRs were identified. We identified 4052 DMGs, including 2855 DMGs that harbored DMRs in gene body regions (from transcription start site (TSS) to transcription end site (TES)) and 1197 DMGs containing DMRs in promoter regions (Additional file 5: Table S3).

### Correlation between DNA methylation and gene expression

**Fig. 3:**
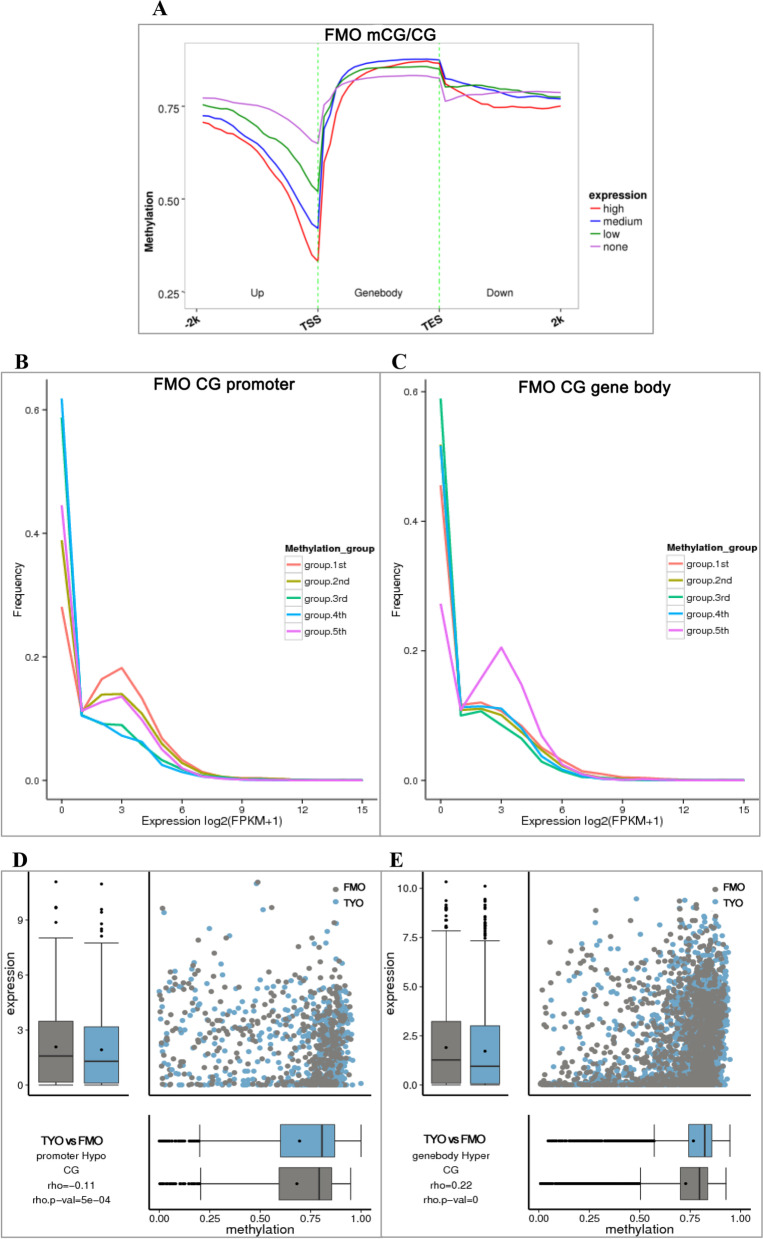
DNA methylation and mRNA expression of genes in different categories. **A** The methylation levels of different gene groups (with different mRNA expression levels) in the five-month-old group. Genes were divided into four groups according to the mRNA expression levels: none, low, medium, and high, and the DNA methylation levels in different groups were compared. **B** mRNA expression levels of different gene categories (with different methylation levels) in promoter regions of the five-month-old group. Genes were classified into five categories based on the methylation levels, from the bottom 20% to the top 20%, corresponding to the 1st to 5th groups, and the mRNA expression levels were compared. **C** mRNA expression levels of different gene categories (with different methylation levels) in gene body regions of the five-month-old group. **D** Correlation between DNA methylation and gene expression of promoter hypo-DMGs. **E** Correlation between DNA methylation and gene expression of gene body hyper-DMGs

The gene expression profiles of the two groups were investigated by RNA-seq using the corresponding samples. Three replicates were performed for each group, and six libraries were obtained. All libraries were sequenced on an Illumina X Ten platform to generate 150 bp paired-end reads. Each library generated ≥7.5 GB of clean data and showed Q30 ≥ 94% (Table 2), implying sufficient quality of RNA-seq data. RNA-seq data were deposited in the SRA at NCBI (accession number: PRJNA634937). To analyze the correlation between DNA methylation and gene expression, the DNA methylation levels and gene expression levels of the gene body regions, 5′ upstream and 3′ downstream, in the two fish groups were analyzed. We first divided the genes into four groups according to their mRNA expression levels: none (unexpressed genes, FPKM < 1), low (FPKM < bottom 25% of expressed genes), medium (bottom 25% of expressed genes ≤ FPKM < top 25% expressed genes), and high (FPKM ≥ top 25% expressed genes). The DNA methylation levels in the different groups were compared. In general, the methylation levels in the four groups began to decrease from 2 kb upstream of the TSS of the genes but increased downstream of the TSS. In the 5′ upstream region, the genes with high mRNA expression levels showed the lowest DNA methylation levels, whereas the unexpressed genes displayed the highest methylation levels (Fig. 3A and Additional file 2: Fig. S2A), suggesting a negative correlation between promoter methylation and gene expression. Nevertheless, the correlation between DNA methylation of the gene body or 3′ downstream and mRNA expression is complex. Moreover, we also classified genes into five categories based on their methylation levels, from the bottom 20% to the top 20%, corresponding to the 1st to 5th groups, and the mRNA expression levels of these groups were investigated. The 1st group of genes in the promoter regions showed the highest expression levels (Fig. 3B and Additional File 2: Fig. S2B), which further implies that DNA methylation in promoter regions, especially hypomethylation, is negatively associated with gene expression. Interestingly, the 5th genes in the gene body region showed the highest expression levels (Fig. 3C and Additional file 2: Fig. S2C), indicating that DNA methylation in gene body regions, particularly hypermethylation, is positively correlated with gene expression.

**Table 2:** Summary of RNA-seq data

Sample name	duplicates	Raw reads	Clean reads	Clean bases	Error rate(%)	Q20(%)	Q30(%)	GC content(%)
FMO	FMO-a	60,792,852	57,206,000	8.58G	0.01	97.95	94.67	46.47
	FMO-b	65,407,362	62,369,440	9.36G	0.01	97.84	94.44	46.59
	FMO-c	64,628,348	61,537,126	9.23G	0.01	97.76	94.28	46.61
TYO	TYO-a	53,314,238	50,037,204	7.51G	0.01	97.85	94.49	46.52
	TYO-b	57,741,144	54,693,810	8.2G	0.01	97.86	94.5	46.28
	TYO-c	56,659,486	53,821,798	8.07G	0.01	97.85	94.45	47.49

Furthermore, the DMRs obtained by WGBS were selected, and the relationship between DNA methylation levels and mRNA expression levels was analyzed using Spearman’s rank correlation method [21]. For the DMRs in the promoter regions, both hyper-DMRs and hypo-DMRs displayed a slightly negative correlation between the DNA methylation levels and mRNA expression levels, especially promoter hypo-DMRs (Fig. 3D and Additional file 2: Fig. S2D). However, for the DMRs in the gene body regions, only the hyper-DMRs showed a slightly positive correlation (r = 0.22, *p* < 0.05) (Fig. 3E), whereas no correlation was observed in the hypo-DMRs (*p* > 0.05) (Additional file 2: Fig. S2E). These findings further suggest that hypomethylation in promoter regions and hypermethylation in gene body regions are associated with gene expression.

### Functional enrichment analysis of DMGs

**Fig. 4:**
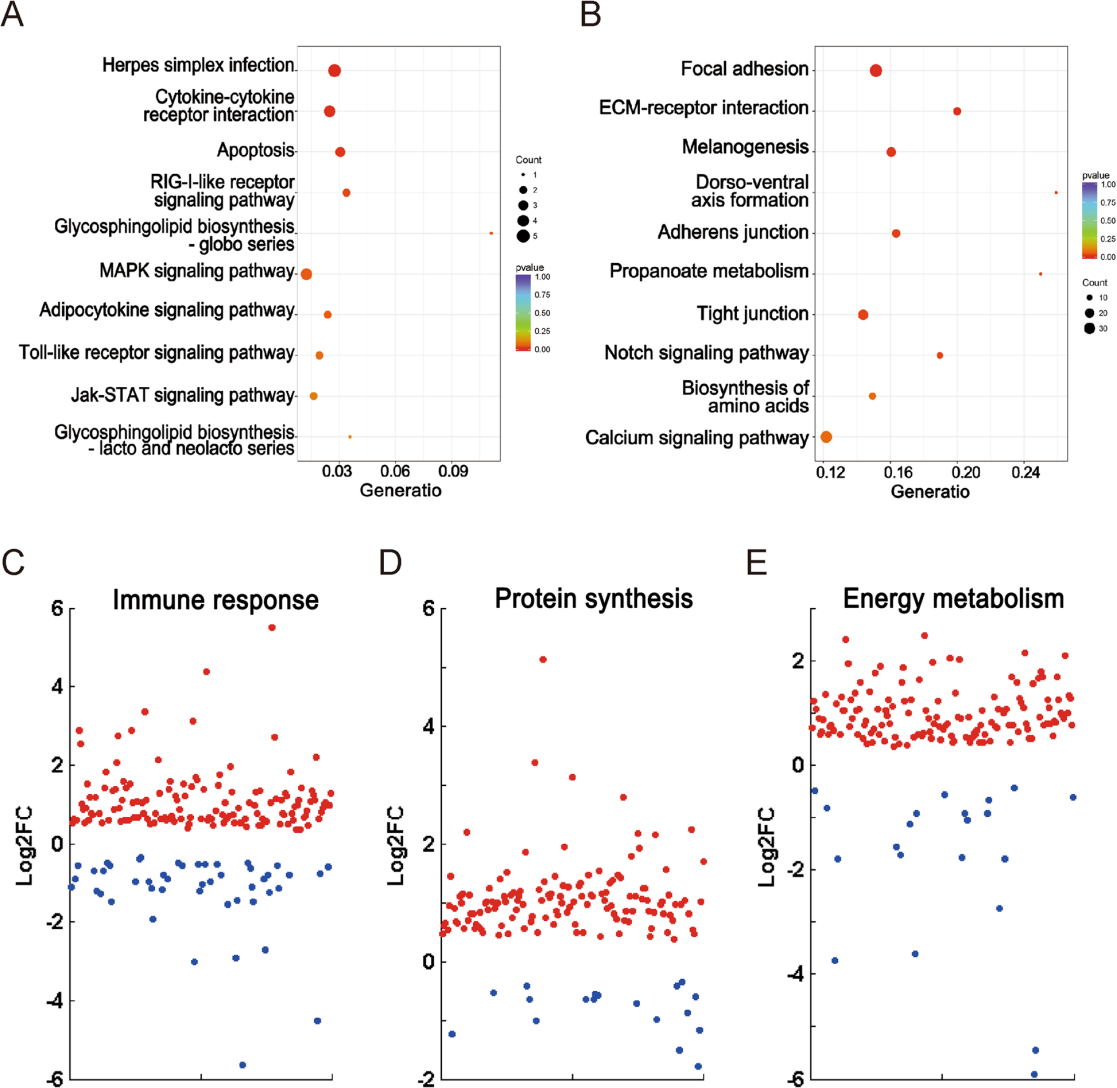
Enrichment analyses of DMGs in different categories and mRNA expression profiles of genes in different categories. **A** KEGG enrichment analyses of DMGs in promoter hypo-DMGs. **B** KEGG enrichment analyses of DMGs in gene body hyper-DMGs. **C** Scatterplots of genes involved in the immune response. **D** Scatterplots of genes involved in protein synthesis. **E** Scatterplots of genes involved in energy metabolism. The red dots represent genes upregulated in TYO fish, while the blue dots represent genes downregulated in FMO fish

Due to hypomethylation in promoter regions, and hypermethylation in gene body regions is associated with gene expression. Therefore, we performed an enrichment analysis for promoter hypo-DMGs (hypomethylated DMGs in promoter regions) and gene body hyper-DMGs (hypermethylated DMGs in gene body regions). GO enrichment analysis revealed that promoter hypo-DMGs were not significantly enriched in any GO terms (corrected *P* value = 1), whereas the gene body hyper-DMGs were enriched in GO terms related to transcription, biosynthesis, and metabolic processes, such as regulation of transcription (DNA-templated), regulation of nucleic acid-templated transcription, regulation of RNA biosynthetic process, regulation of RNA metabolic process, and regulation of cellular macromolecule biosynthetic process. The top ten significantly enriched GO terms are listed in Table 3. In addition, KEGG enrichment analysis was performed. Interestingly, some typical immune-related pathways, such as herpes simplex infection, cytokine-cytokine receptor interaction, RIG-I-like receptor signaling pathway, and Toll-like receptor signaling pathway, were significantly enriched in promoter hypo-DMGs (Fig. 4A). The gene body hyper-DMGs were enriched in KEGG pathways, such as focal adhesion, ECM-receptor interaction, melanogenesis, dorso-ventral axis formation, adherens junction, and some pathways involved in metabolism and biosynthesis (propanoate metabolism and biosynthesis of amino acids) (Fig. 4B). Detailed information on the KEGG enrichment analysis is shown in Additional file 6: Table S4.

**Table 3:** GO enrichment analysis of promoter hypo-DMGs and gene body hyper-DMGs (top ten terms)

Categories	GO terms	Corrected *P* Value	DMR genes
promoter hypo-DMGs	regulation of cell shape	1	2
	regulation of cell morphogenesis	1	2
	electron transporter, transferring electrons within the cyclic electron transport pathway of photosynthesis activity	1	2
	tumor necrosis factor receptor binding	1	2
	tumor necrosis factor receptor superfamily binding	1	2
	DNA-directed DNA polymerase activity	1	3
	immune system process	1	6
	electron carrier activity	1	4
	photosynthesis, light reaction	1	2
	regulation of anatomical structure morphogenesis	1	2
gene body hyper-DMGs	regulation of transcription, DNA-templated	1.44E-07	262
	regulation of nucleic acid-templated transcription	1.44E-07	262
	regulation of RNA biosynthetic process	1.44E-07	262
	regulation of RNA metabolic process	1.44E-07	262
	regulation of nucleobase-containing compound metabolic process	1.44E-07	263
	regulation of cellular macromolecule biosynthetic process	1.55E-07	265
	regulation of macromolecule biosynthetic process	1.55E-07	265
	regulation of cellular biosynthetic process	1.55E-07	265
	regulation of biosynthetic process	1.55E-07	265
	regulation of nitrogen compound metabolic process	1.55E-07	265

### Gene expression patterns of promoter hypo-DMGs and gene body hyper-DMGs

Hypomethylation in the promoter regions was thought to be associated with transcriptional activation (Fig. 3B), and KEGG enrichment analysis showed that promoter hypo-DMGs were enriched in classical innate immune pathways (Fig. 4A). It is well known that the immune response plays an important role in host defense against pathogen infection. Therefore, we analyzed the expression profiles of immune-related genes between two groups, and the results showed that most of them (74.87%) were upregulated in TYO fish (Fig. 4C). Moreover, it was proposed that gene body hypermethylation involved in gene expression (Fig. 3C) and gene body hyper-DMGs were enriched in pathways related to transcription, biosynthesis, and metabolism (Table 3). Protein synthesis and energy metabolism also benefit organisms that are resistant to viral infection. We therefore investigated the mRNA expression patterns of genes involved in protein biosynthesis and energy metabolism to further confirm this hypothesis. As expected, most of the genes involved in biosynthesis (88.31%) and energy metabolism (86.96%) were also upregulated in TYO fish (Fig. 4D, E).

### Confirmation of WGBS data by BS-PCR and qPCR

**Fig. 5:**
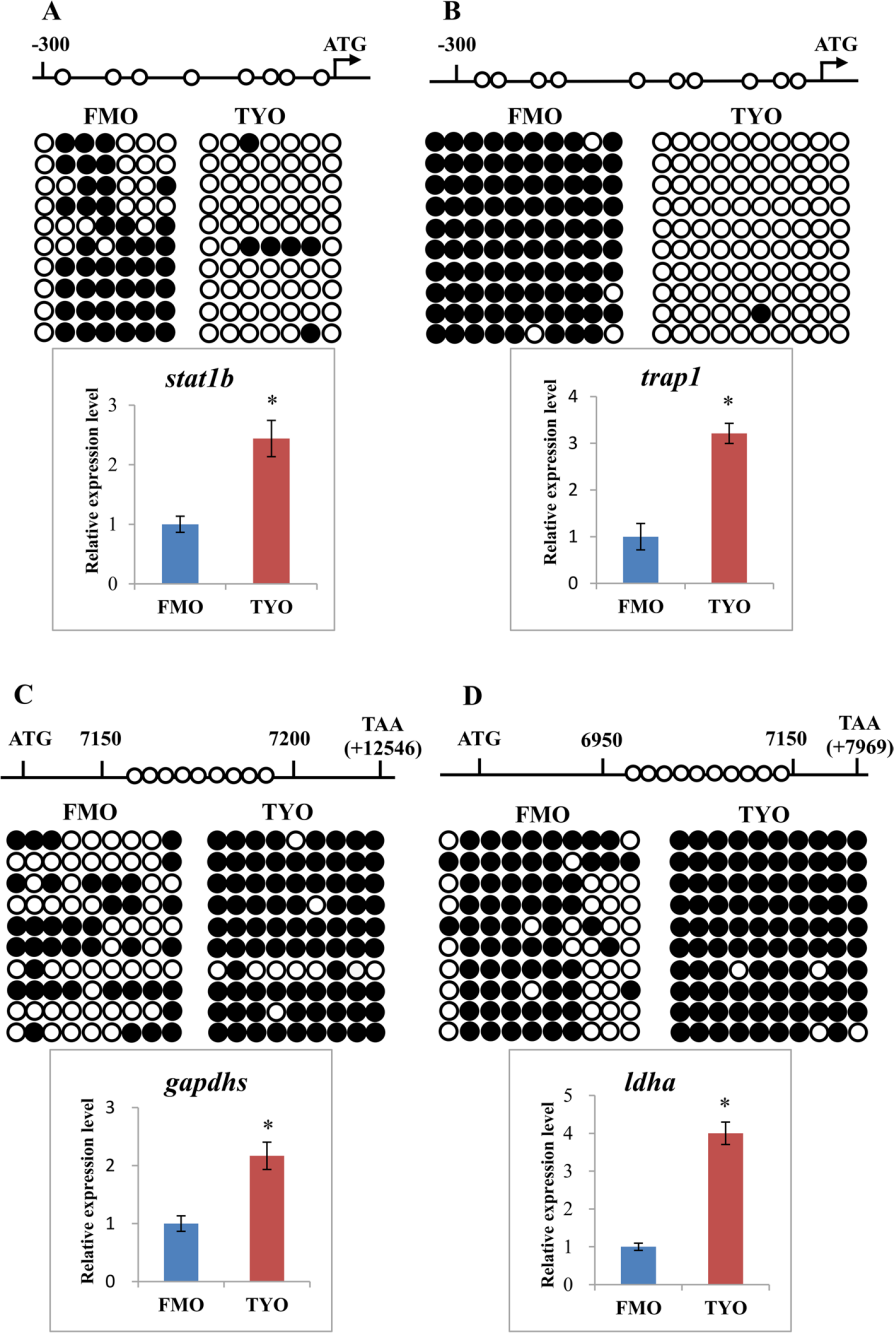
Confirmation of WGBS data by BS-PCR and qPCR. **A** DNA methylation and gene expression profiles in the promoter region of *stat1b* gene. **B** DNA methylation and gene expression profiles in the promoter region of *trap1* gene. **C** DNA methylation and gene expression profiles in the gene body region of *gapdh* gene. **D** DNA methylation and gene expression profiles in the gene body region of *idha* gene. Top panels: schematic diagram of promoter regions or gene body regions of four genes. The circles indicate the candidate CpG loci in the DMRs. Middle panels: Validation of CpG methylation by BS-PCR. Fifteen subclones from each group were selected for the BSP analysis. The solid circles represent the methylated CpG sites, and the open circles indicate the unmethylated CpG sites. Bottom panels: Comparison of gene expression levels between two groups by qPCR

These results indicated that DNA methylation in promoter regions was negatively associated with gene expression, and that promoter hypo-DMGs were enriched in immune-related pathways (Fig. 4A). Therefore, two immune-related genes, signal transducer and activator of transcription 1b (*stat1b*) and TNF receptor-associated protein 1 (*trap1*), were selected for further confirmation. The CpG loci of candidate DMRs in the gene promoter were amplified by bisulfite sequencing PCR (BS-PCR) and mRNA expression levels were determined by qPCR. As shown in Fig. 5A and B, BS-PCR showed that the DNA methylation level of the promoter regions of two immune-related genes in FMO fish was higher than that in TYO fish, while the mRNA expression levels presented opposite trends, implying that DNA methylation in promoter regions was negatively associated with gene expression. Moreover, two genes involved in protein biosynthesis and energy metabolism, glyceraldehyde-3-phosphate dehydrogenase glyceraldehyde-3-phosphate dehydrogenase, spermatogenic (*gapdhs*), and lactate dehydrogenase A (*ldha*), were selected, and the CpG loci of DMRs in the gene body region were amplified for BS-PCR verification and mRNA expression levels were determined by qPCR. As expected, both of the two genes showed higher DNA methylation levels in TYO fish than in FMO fish, and the mRNA expression also showed similar trends (Fig. 5C and D), further suggesting a positive correlation between gene body DNA methylation and gene expression.

### The antiviral effects of metabolism-related genes

**Fig. 6:**
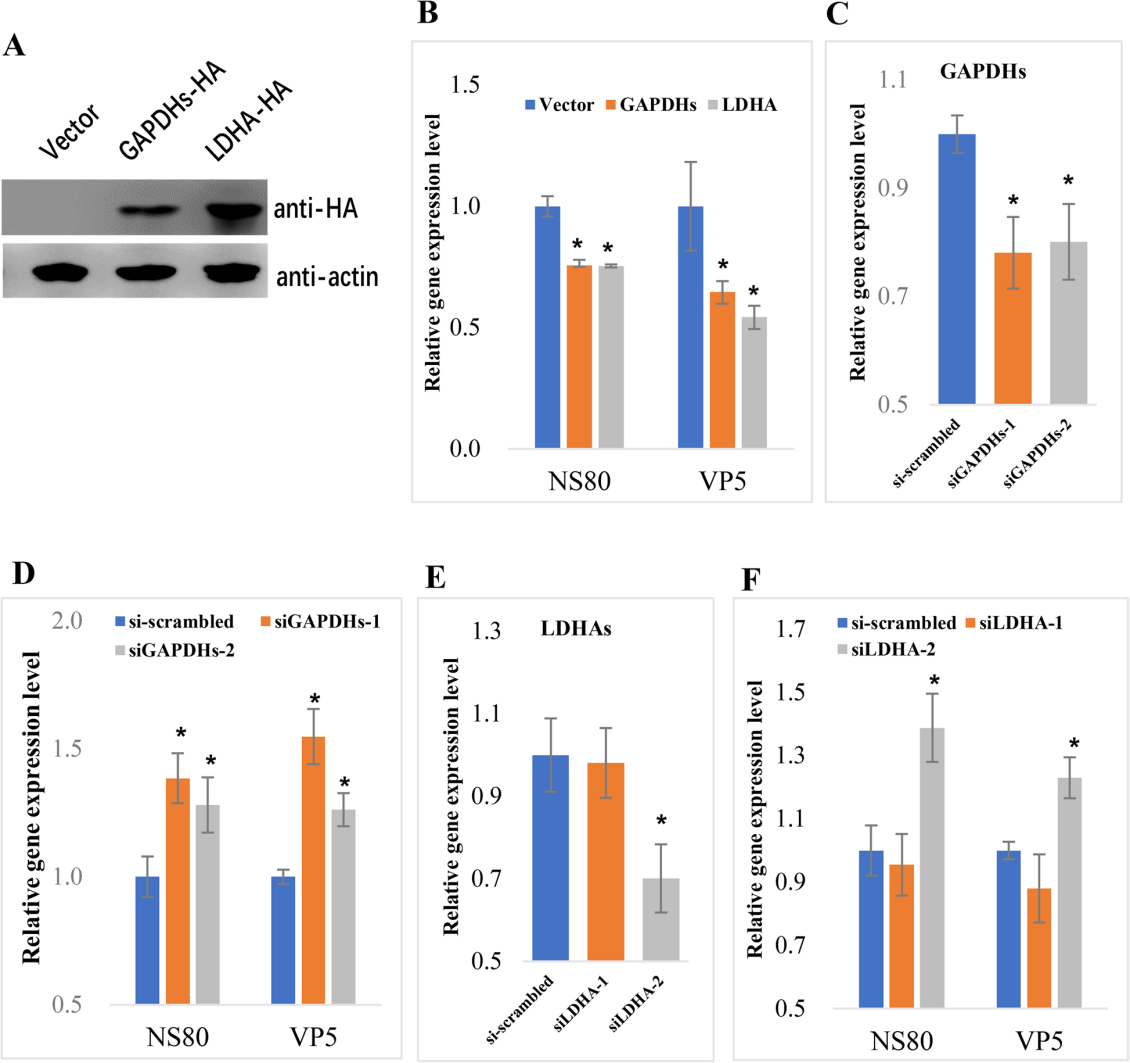
The antiviral effects of two metabolism-related genes. **A** Western blotting confirmation of the overexpression of two genes in transfected cells. Cells were transfected with the corresponding plasmids for 24 h and harvested for western blotting analysis. Rabbit anti-HA antibody and goat anti-rabbit IgG were used as the primary and secondary antibodies, respectively. **B** The relative copy number of genes encoding GCRV nonstructural protein NS80 and structural protein VP5 in cells transfected with different plasmids. **C** RT-qPCR confirm the knockdown of *gapdhs* by siRNAs. Cells were transfected with the corresponding siRNA for 24 h and harvested for RT-qPCR analysis. **D** The relative copy number of genes encoding NS80 and VP5 in cells transfected with different siRNAs. **E** RT-qPCR confirm the knockdown of *ldha* by siRNAs. **F** The relative copy number of genes encoding NS80 and VP5 in cells transfected with different siRNAs. Significant differences (*P* < 0.05) between the control and treated groups are indicated with asterisks (*)

The above results showed that most of the genes involved in the immune response and energy metabolism were upregulated in TYO fish when compared with FMO fish. It is well known that immune-related genes play important roles in host defense against pathogen infection; however, the role of metabolism-related genes during viral infection is unclear. Therefore, two metabolism-related genes, *gapdhs* and *ldha*, were selected for overexpression or knockdown to investigate their roles in GCRV infection. As shown in Fig. 6, after overexpression of two genes in grass carp ovary (GCO) cells (Fig. 6A), the relative copy number of GCRV nonstructural protein NS80 and structural protein VP5 was significantly lower than that in the negative control (Fig. 6B). In contrast, when the expression of the two genes was knockdown by specific siRNAs (Fig. 6C and E), the relative copy numbers of NS80 and VP5 were significantly higher than those in the negative control (Fig. 6D and F). Collectively, these results indicated the antiviral effects of two metabolism-related genes.

## Discussion

Age-dependent viral susceptibility has been reported in several fish species. Nervous necrosis virus (NNV) infection in barramundi (*Lates calcarifer*) causes nervous necrosis at 3 and 4 weeks of age, but develops subclinical symptoms when fish age is more than 5 weeks [22]. Spring viremia of carp virus (SVCV) challenge in North American fish species revealed that younger fish were more vulnerable to SVCV infection than older fish [23]. In our previous study, we found that grass carp showed age-dependent susceptibility to reovirus, and the immune response level or biosynthesis and metabolism levels may account for this phenomenon [9]. Understanding the underlying mechanisms of different immune responses or metabolism patterns in fish of different ages will benefit disease-resistant breeding programs.

DNA methylation, the most widely understood type of epigenetic modification, has been reported to play a crucial role in transcriptional regulation [24, 25]. It is generally accepted that DNA hypomethylation in gene promoters is linked to transcriptional activation, whereas hypermethylation in these regions is correlated with transcriptional repression [26, 27]. Nevertheless, some evidence also shows that promoter hypermethylation appears to be associated with high transcriptional activity [28], indicating that DNA methylation contributes to transcriptional regulation in a more complex and dynamic manner. DNA methylation has been reported to be correlated with the disease resistance traits of Chinese tongue sole and Nile tilapia [29, 30]. Therefore, we hypothesized that DNA methylation may be involved in age-dependent viral susceptibility of grass carp. Here, we report a genome-wide survey of the DNA methylation status of spleens from susceptible FMO grass carp versus resistant TYO grass carp to reveal the potential epigenetic mechanisms of this phenomenon.

Interestingly, we found that the general DNA methylation pattern of grass carp was consistent with that of other species [20, 31–33]. For example, most mC sites occurred in the CG context, with relatively low ML in the promoter or 5′ UTR regions and high ML in repeat regions, and a negative correlation between methylation of promoter or 5′ UTR regions and gene expression. These results indicate a conserved role for DNA methylation during evolution. Interestingly, we showed that genes with the highest DNA methylation levels in the gene body regions had the highest mRNA expression levels in both groups (Fig. 3C and Additional file 2: Fig. S2C), suggesting that hypermethylation in gene body regions was positively correlated with mRNA expression. The positive correlation was further confirmed in two biosynthesis- or metabolism-related genes using BS-PCR and qPCR (Fig. 5). Several literatures also revealed the positive correlation between gene body methylation and gene expression [34, 35]. Moreover, it was reported that gene body hypermethylation is frequent occurred in constitutively expressed genes, whereas least frequent in variable expressed genes [36, 37]. Enrichment analysis revealed that gene body hyper-DMGs were involved in genes related to transcription, biosynthesis, and metabolism, all of them are constitutively expressed genes. It was proposed that the function of gene body hypermethylation is most likely homeostatic, which may maintain gene expression in response to developmental and/or environmental stresses [38]. Therefore, the high gene body methylation levels in TYO fish may imply that the TYO fish was more stable than FMO fish after GCRV infection, which may be one of the reasons for age-dependent viral susceptibility in grass carp.

Correlation analysis revealed DNA hypomethylation in promoter regions and hypermethylation in gene body regions associated with gene expression. Therefore, we performed enrichment analysis for promoter hypo-DMGs and gene body hyper-DMGs. The results suggested that promoter hypo-DMGs were significantly enriched in classical immune-related pathways (Fig. 4A), whereas the gene body hyper-DMGs were enriched in terms related to transcription, biosynthesis, and metabolism (Table 3 and Fig. 4B). Coincidently, RNA-seq using the corresponding samples also revealed that most of the genes participating in the immune response, biosynthesis, and metabolism were upregulated in resistant TYO fish (Fig. 4C-E). The role of immune-related genes in viral infection is well documented [39], whereas the functions of metabolism-related genes is still unclear. Gene function analysis by gene overexpression or knockdown indicated the antiviral effects of metabolism-related genes. The upregulation of metabolism-related genes indicated high metabolism levels in TYO fish, which was beneficial for host defense against pathogen infection. Previous studies have also suggested that elevated host metabolism promotes resistance to pathogen infection in fish [9, 40, 41]. Therefore, based on these results, we conclude that different immune response levels or different biosynthesis and metabolism levels between FMO and TYO fish may be mediated by DNA methylation.

## Conclusion

In conclusion, genome-wide variations in DNA methylation and gene expression in grass carp of different ages were investigated. We identified 6214 DMRs and 4052 DMGs, with most DMRs and DMGs showing hypermethylation patterns in TYO fish. Correlation analysis indicated that DNA hypomethylation in promoter regions and hypermethylation in gene body regions are involved in transcriptional activation. DNA methylation and gene expression variations in genes involved in immune response, biosynthesis, and energy metabolism may contribute to age-dependent viral susceptibility in grass carp.

## Methods

### Experimental fish, GCRV challenge experiment, and sample collection

A total of 500 fish, containing 250 full-sib FMO and 250 full-sib TYO grass carp, representing fish sensitive and resistant to GCRV infection, respectively, were used in the study. All fish were bred at the Guan Qiao Experimental Station, Institute of Hydrobiology, Chinese Academy of Sciences (CAS), and acclimatized in aerated fresh water at 26–28 °C for one week before processing. To ensure that the grass carp were not previously exposed to GCRV, the fish were randomly selected for PCR detection of GCRV using GCRV-specific primers (Additional file 7: Table S5). The GCRV-free fish were fed commercial feed twice daily, and the water was exchanged daily.

After no abnormal symptoms were observed in the two groups, 200 fish from each group were subjected to a viral challenge experiment. Fish were infected with GCRV (GCRV subtype II, 2.97 × 10^3^ RNA copies/μL) at a dose of 20 μL per gram of body weight by intraperitoneal injection. The injected fish were carefully monitored, and the number of dead fish in each group was counted daily. The experiment was concluded, and the total mortality was calculated when no mortality was recorded for seven consecutive days.

The remaining fish, which were not included in the viral challenge experiment, were used for sample collection. The sex ratio (male: female) of the sampled fish was approximately 1:1. In each group, 15 fish containing three biological replicates (*n* = 5 for each biological duplicate) were collected. Spleen samples were removed rapidly for analysis after the fish were anesthetized and euthanized using MS-222. The obtained samples were stored at − 80 °C until DNA or RNA extraction.

### Whole-genome bisulfite sequencing

Genomic DNA was extracted from the spleen using the DNeasy Blood & Tissue Kit (Qiagen, Hilden, Germany). DNA purity and concentration were measured using a NanoPhotometer® spectrophotometer (IMPLEN, USA) and a Qubit® 2.0 Flurometer (Life Technologies, USA). DNA of sufficiently high quality was used for library construction. A total of 5.2 μg DNA spiked with 26 ng λDNA was fragmented by sonication to 200–300 bp with Covaris S220, followed by end repair and adenylation. Cytosine-methylated barcodes were ligated to sonicated DNA, according to the manufacturer’s instructions. These DNA fragments were then treated twice with bisulfite using an EZ DNA Methylation-Gold™ Kit (Zymo Research, USA), and the resulting single-stranded DNA fragments were PCR-amplified using KAPA HiFi HotStart Uracil + ReadyMix. The library concentration was quantified using a Qubit® 2.0 Fluorometer and quantitative PCR, and the insert size was assayed using an Agilent Bioanalyzer 2100 system. Libraries were sequenced on an Illumina HiSeq X Ten platform, and 150 bp paired-end reads were generated.

### Data analysis

Raw data reads in fastq format were initially processed through a series of quality control (QC) procedures using in-house Perl scripts to obtain clean data. QC standards were as follows: (1) reads with ≥10% unidentified nucleotides (N) were removed; (2) reads with > 50% bases with phred quality < 5 were removed; (3) reads with > 10 nt aligned to the adapter were removed, allowing ≤10% mismatches; and (4) putative PCR duplicates generated by PCR amplification in the library construction process (reads 1 and 2 of two paired-end reads that were completely identical) were removed. The Q20, Q30, and GC content of the clean data were calculated, and all downstream analyses were performed using high-quality clean data. The reference genome of grass carp and the clean reads were transformed into bisulfite-converted sequences (C-to-T and G-to-A converted). Bismark software (version 0.16.3) was used to align the bisulfite-treated reads to the reference genome [42]. Sequence reads that produced the unique best alignment from the two alignment processes (original top and bottom strands) were then compared to the normal genomic sequence, and the methylation state of all cytosine positions in the read was inferred. The same reads aligned to the same regions of the genome in the two alignment processes were regarded as duplicate reads. The sequencing depth and coverage were summarized using duplicate reads. The methylation extractor results were transformed into bigWig format for visualization using the IGV browser. The sodium bisulfite non-conversion rate was calculated as the percentage of cytosines sequenced at cytosine reference positions in the lambda genome.

To calculate the methylation level of the sequence, we divided it into multiple bins with a size of 10 kb. The sum of methylated and unmethylated read counts in each window/bin was calculated. The methylation level (ML) for each window or C site shows the fraction of methylated Cs (mC) and is defined as ML = reads (mC)/reads (mC + umC), where umC is unmethylated Cs. The calculated ML was further corrected using the bisulfite nonconversion rate according to previous studies [43].

### Differentially methylated regions analysis

Differentially methylated regions (DMRs) analysis of the two groups/conditions was performed using DSS software [44]. The core of DSS is a new dispersion shrinkage method for estimating the dispersion parameter from the gamma-Poisson or beta-binomial distributions. A DSS possesses three characteristics for detecting DMRs. First, spatial correlation and proper utilization of information from neighboring cytosine sites can help improve the estimation of methylation levels at each cytosine site and hence improve DMRs detection. Second, the read depth of cytosine sites provides information on precision that can be exploited to improve statistical tests for DMRs detection. Finally, the variance among biological replicates provided the information necessary for a valid statistical test to detect DMRs. Differentially methylated genes (DMGs) were identified as genes whose gene body regions (from TSS to TES) or promoter regions (upstream 2 kb from the TSS) overlapped with the DMRs. Gene ontology (GO) and Kyoto Encyclopedia of Genes and Genomes (KEGG) enrichment analyses of DMGs were performed using the GOseq R package and KOBAS software [45, 46].

### RNA -seq

RNA from the spleens was isolated using TRIzol reagent (Invitrogen, USA) according to the manufacturer’s protocol. RNA of sufficiently high quality was used for library construction. Sequencing libraries were generated using the NEBNext Ultra RNA Library Prep Kit for Illumina (New England Biolabs, USA), following the manufacturer’s protocol. Libraries were sequenced on an Illumina X Ten platform, and 150 bp paired-end reads were generated. The output raw data were initially processed with in-house Perl scripts to obtain clean data by removing adapter, poly-N, and poor-quality data, as described above. The clean reads were mapped to the reference genome of grass carp using Hisat2 software [47], and gene expression levels were calculated using the FPKM (expected number of fragments per kilobase of transcript sequence per million base pairs sequenced) method [48]. Differential expression analysis of the two groups/conditions was performed using the DESeq package [49]. Genes with an adjusted *P* value < 0.05 (q value < 0.05) in DESeq analysis were assigned as DEGs.

### Global DNA methylation measurement

Genomic DNA from the spleens of FMO and TYO fish was extracted using the traditional phenol–chloroform protocol with RNase treatment [50]. The global DNA methylation level was measured using the MethylFlash™ Global DNA Methylation (5-methylcytosine, 5-mC) ELISA Easy Kit (Epigentek, USA), according to the manufacturer’s protocol. The amount of input DNA for each assay was 100 ng, to ensure optimal quantification. Three replicates were performed for each group. Data are presented as the mean ± standard deviation of three replicates.

### Bisulfite sequencing PCR

Bisulfite sequencing PCR (BS-PCR) was performed to confirm the DNA methylation levels of the DMGs between the two groups. DNA samples were treated with bisulfite using the EZ DNA Methylation Gold™ Kit (Zymo Research, USA), according to the manufacturer’s instructions. DMGs related to immune response or biosynthesis were selected as candidates for further verification. Genomic sequences of the candidate DMGs were extracted from the grass carp draft genome [3] and submitted to the online software MethPrimer [51] to obtain the CpG island region and CpG loci. Specific methylation primers (Additional file 7: Table S5) for BS-PCR were designed using the MethPrimer software to amplify the CpG island region or CpG loci that showed different methylation levels. The lengths of the amplified regions ranged from 150 to 350 bp. Moreover, an outer forward or reverse primer was designed for each amplified region to improve the specificity of the amplification. The obtained DNA fragments were cloned into the pMD18-T vector (TaKaRa, Japan). Fifteen clones from each group were randomly selected for sequencing to evaluate methylation status.

### RT-qPCR

RT-qPCR was used to investigate the mRNA expression levels of DMGs in the two groups. Total RNA was isolated using TRIzol reagent (Thermo Fisher, USA) and first-strand cDNA was obtained using a ReverTra Ace kit (Toyobo, Japan). RT-qPCR was performed using a fluorescence quantitative PCR instrument (Bio-Rad, USA). Each reaction mixture contained 0.8 μL forward and reverse primers (for each primer), 1 μL cDNA template, 10 μL 2× SYBR green master mix (Toyobo, Japan), and 7.4 μL ddH_2_O. DMGs related to immune response or biosynthesis were selected as candidates for RT-qPCR verification. The cDNA sequences of candidate DMGs were extracted from the grass carp genome, and specific primers were designed using the Primer Premier 5software (Additional file 7: Table S5). Three replicates were included for each sample, and β-actin was used as an internal control to normalize the gene expression. The program was as follows: 95 °C for 10 s; 40 cycles of 95 °C for 15 s, 56 °C for 30 s, and 72 °C for 30 s; and melt curve construction. Relative expression levels were calculated using the 2^-△△Ct^ method [52]. The data represent the mean ± standard deviation of three replicates.

### Gene function analysis

Two metabolism-related genes, *gapdhs* and *ldha*, were selected for further study to investigate their roles during GCRV infection. The complete ORF sequences of the two genes were amplified, fused with an HA tag, and then inserted into the pcDNA3.1 vector (Additional file 7: Table S5). The resulting plasmids were transfected into the GCO cells. The empty vector pcDNA3.1 was also transfected at the same time as a negative control. After transfection for 24 h, cells were infected with GCRV at a multiplicity of infection (MOI) of 1. Cells were collected at 24 and 48 h post-infection, and the relative copy number of genes encoding the GCRV nonstructural protein NS80 or structural protein VP5 was determined by RT-qPCR. Moreover, siRNAs specifically targeting these two genes were synthesized to generate the corresponding siRNAs (Additional file 7: Table S5). The siRNA sequences were randomly scrambled and used as negative controls. All siRNAs used in this study were synthesized by GenePharma (Shanghai, China). GCO cells were transfected with the siRNAs and then infected with GCRV at a MOI of 1 24 h after transfection. Cells were harvested 24 and 48 h post-infection, and the relative copy number of genes encoding NS80 or VP5 was determined by RT-qPCR.

### Statistical analysis

Statistical significance between different groups was determined by one-way analysis of variance (ANOVA) and Fisher’s least significant difference (LSD) post-test. Differences were considered statistically significant at *P* < 0.05. *P* < 0.05 was denoted by *.

## Supplementary Information

**Additional file 1: Fig. S1.** Whole-genome bisulfite sequencing of the FMO and TYO fish groups. A and B Pearson’s correlation analysis (A) and principal component analysis (B) of the six samples from the two groups. C and D Distribution of mCs in different genomic regions (C) and in different gene elements (D). E and F Methylation levels (E) and length distribution (F) of DMRs.

**Additional File 2: Fig. S2.** Correlation between DNA methylation and gene expression (A) The methylation levels of different gene groups (with different mRNA expression levels) in the TYO group. B The mRNA expression levels of different gene categories (with different methylation levels) in the promoter region of the TYO group. C mRNA expression levels of different gene categories (with different methylation levels) in the gene body regions of the TYO group. D Correlation between DNA methylation levels and gene expression levels of promoter hyper-DMGs. E Correlation between DNA methylation levels and gene expression levels of gene body hypo-DMGs.

**Additional file 3: Table S1.** Methylated cytosines percentage of different context

**Additional file 4: Table S2.** Detailed information on DMRs identified between the two fish groups.

**Additional file 5: Table S3.** Detailed information on the DMGs between the two fish groups.

**Additional file 6: Table S4.** Detailed information on KEGG enrichment analysis of DMGs.

**Additional file 7: Table S5.** Oligonucleotide sequences used in the study.

## Data Availability

The WGBS and RNA-seq data generated in this study were deposited in the Sequence Read Archive (SRA) at the National Center for Biotechnology Information (NCBI) under accession numbers PRJNA638254 and PRJNA634937. Other data generated or analyzed during this study are included in this published article.

## Abbreviations

CAS: Chinese Academy of Sciences
DEGs: Differentially expressed genes
DMGs: Differentially methylated genes
DMRs: Differentially methylated regions
FMO: Five months old
GCRV: Grass carp reovirus
IAV: Influenza virus
mCs: Methylated cytosines
ML: Methylation level
NCBI: National Center for Biotechnology Information
NNV: Nervous necrosis virus
PRV: Pseudorabies virus
SRA: Sequence Read Archive
SVCV: Spring viremia of carp virus
TES: Transcription end site
TSS: Transcription start site
TYO: Three year old
UTR: Untranslated regions
WGBS: Whole-genome bisulfite sequencing
