# Supplementary figures and images for: Genome-wide DNA methylation reveals potential epigenetic mechanism of age-dependent viral susceptibility in grass carp
Source: Immun Ageing. 2022 Jun 2;19:28. doi: 10.1186/s12979-022-00285-w (PMC9161582; doi:10.1186/s12979-022-00285-w)

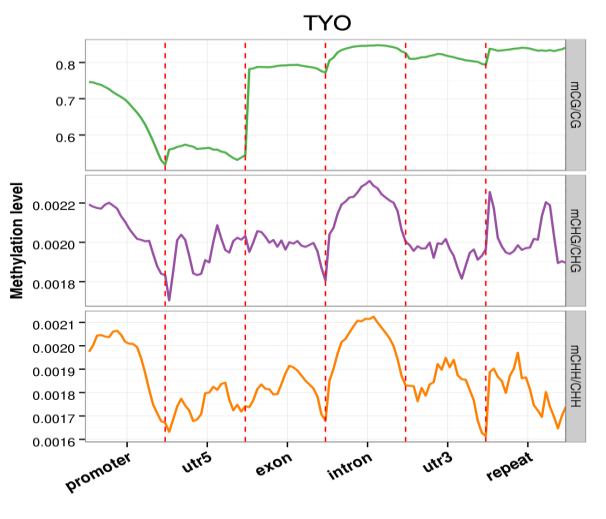

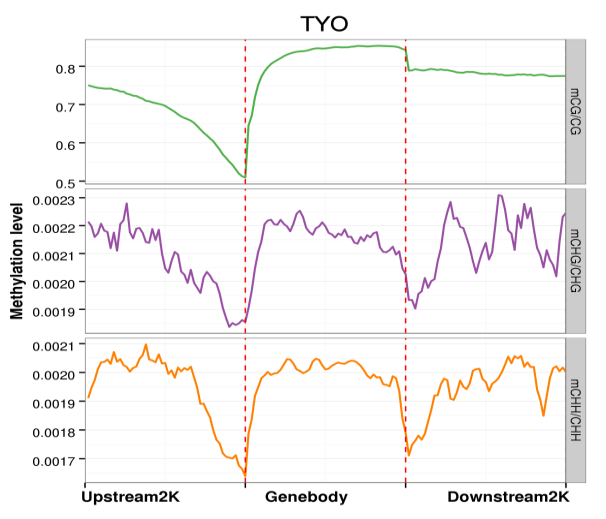

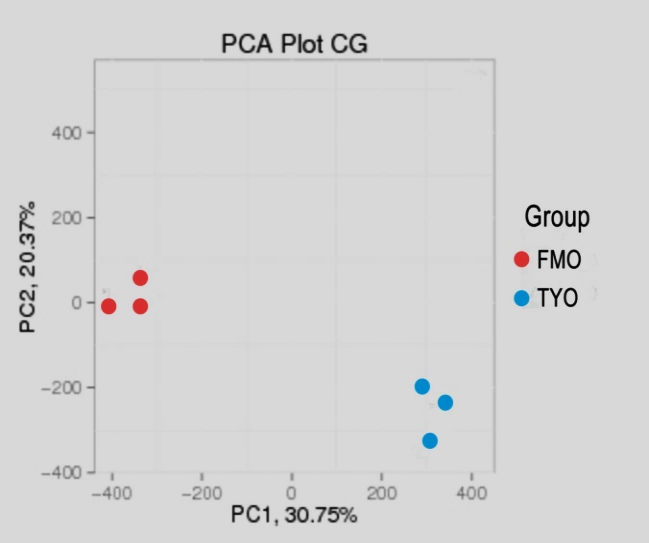

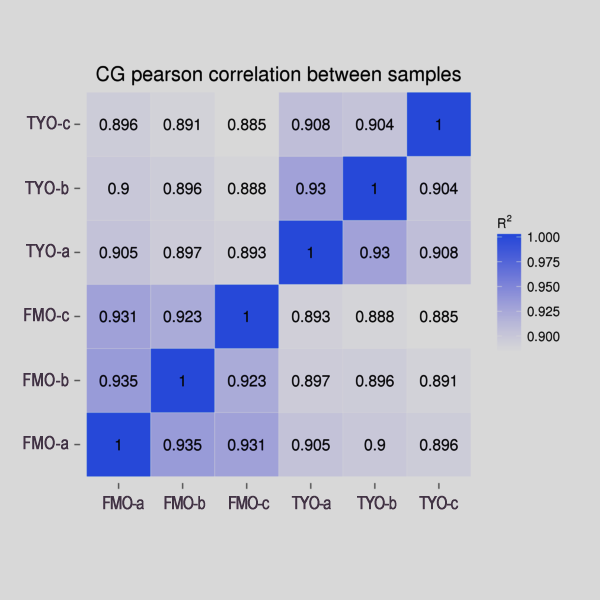


**C**

**D**

**B**

**A**


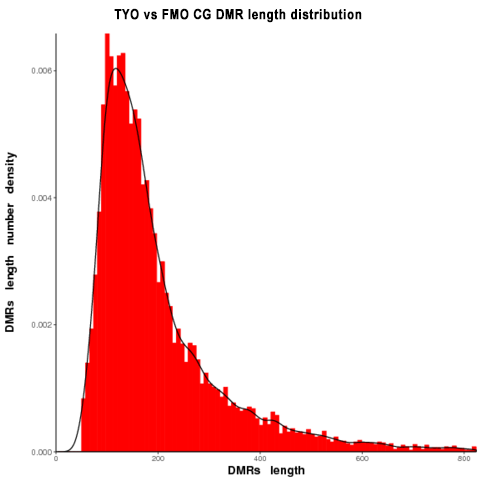

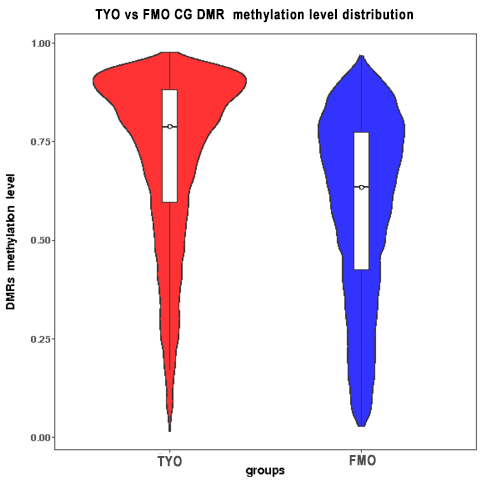


**F**

**E**

**Additional file 1: Figure S1**

Supplement: Supplementary file 1 — Additional file 1: Fig. S1. Whole-genome bisulfite sequencing of the FMO and TYO fish groups. A and B Pearson’s correlation analysis (A) and principal component analysis (B) of the six samples from the two groups. C and D Distribution of mCs in different genomic regions (C) and in different gene elements (D). E and F Methylation levels (E) and length distribution (F) of DMRs. [file 12979_2022_285_MOESM1_ESM.docx]

**A**


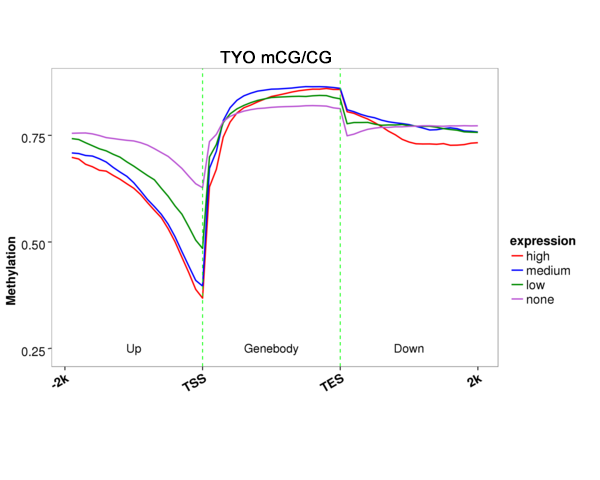


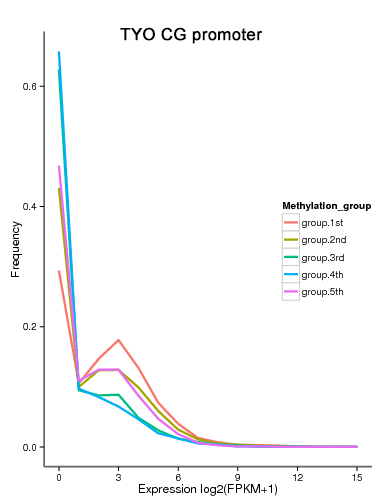

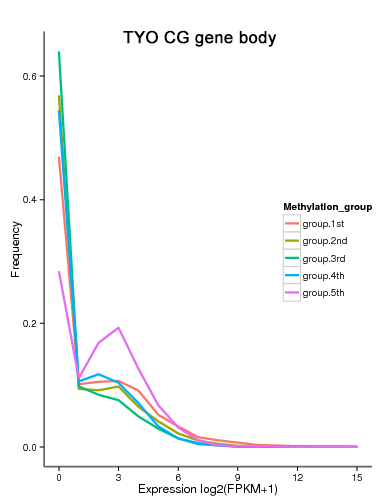


**C**

**B**


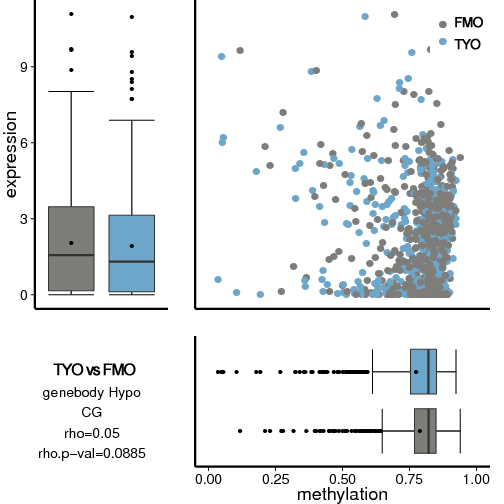


**E**

**D**

**Additional file 2: Figure**
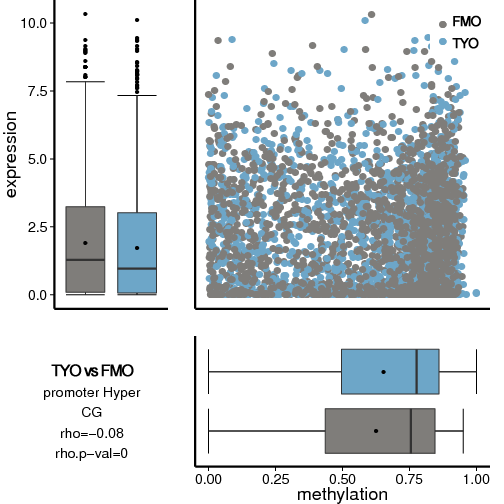
**S2**

Supplement: Supplementary file 2 — Additional File 2: Fig. S2. Correlation between DNA methylation and gene expression (A) The methylation levels of different gene groups (with different mRNA expression levels) in the TYO group. B The mRNA expression levels of different gene categories (with different methylation levels) in the promoter region of the TYO group. C mRNA expression levels of different gene categories (with different methylation levels) in the gene body regions of the TYO group. D Correlation between DNA methylation levels and gene expression levels of promoter hyper-DMGs. E Correlation between DNA methylation levels and gene expression levels of gene body hypo-DMGs. [file 12979_2022_285_MOESM2_ESM.docx]
