# Supplementary material for: Genome-wide DNA methylation reveals potential epigenetic mechanism of age-dependent viral susceptibility in grass carp
Source: Immun Ageing. 2022 Jun 2;19:28. doi: 10.1186/s12979-022-00285-w (PMC9161582; doi:10.1186/s12979-022-00285-w)
Supplement: Supplementary file 3 — Additional file 3: Table S1. Methylated cytosines percentage of different context [file 12979_2022_285_MOESM3_ESM.docx]

**Additional file 3: Table S1 Methylated cytosines percentage of different context**

| **Sample name** | **duplicates** | **mC**  **percent(%)** | **mCpG percent(%)** | **mCHG percent(%)** | **mCHH**  **percent(%)** |
| --- | --- | --- | --- | --- | --- |
| FMO | FMO-a | 5.33% | 54.88% | 0.09% | 0.11% |
|  | FMO-b | 4.81% | 49.47% | 0.09% | 0.10% |
|  | FMO-c | 4.84% | 49.91% | 0.08% | 0.08% |
|  | average | 4.99% | 51.42% | 0.09% | 0.10% |
| TYO | TYO-a | 4.96% | 51.02% | 0.09% | 0.10% |
|  | TYO-b | 4.66% | 48.02% | 0.08% | 0.09% |
|  | TYO-c | 4.77% | 49.17% | 0.08% | 0.09% |
|  | average | 4.80% | 49.40% | 0.08% | 0.09% |
