# Supplementary material for: Genome-wide DNA methylation reveals potential epigenetic mechanism of age-dependent viral susceptibility in grass carp
Source: Immun Ageing. 2022 Jun 2;19:28. doi: 10.1186/s12979-022-00285-w (PMC9161582; doi:10.1186/s12979-022-00285-w)
Supplement: Supplementary file 7 — Additional file 7: Table S5. Oligonucleotide sequences used in the study. [file 12979_2022_285_MOESM7_ESM.docx]

**Additional file 7: Table S5 Oligonucleotide sequences used in the study**

| **primers** | **Sequences (5’ to 3’ )** | **usage** |
| --- | --- | --- |
| GCRV-F | AGCGCAGCAGGCAATTACTATCT | GCRV detection |
| GCRV-R | ATCTGCTGGTAATGCGGAACG |  |
| *stat1b*-BSP-F | TTAAAATAATTTTTTGAGTTAAAATAGTAG | BS-PCR of *stat1b* |
| *stat1b*-BSP-R | TAATCCAACTCTTTTTATTATTCCATC |  |
| *stat1b*-BSP-outer-R | CCTACACACTCTTCTTCAACTTATTAAC |  |
| *trap1*-BSP-F | AAAAGAAGATTAAGTTGTTAAGTGAGTTTT | BS-PCR  of *trap1* |
| *trap1*-BSP-R | TACTATAATTTAACCTATAAAAAACTAAAAAC |  |
| *trap1*-BSP-outer-R | CTTAATAATTAAATCATTTCTACTTCTACTAC |  |
| *gapdhs*-BSP-outer-F | GAAGTTGGTTGTTTTTAGTTAGTTGTG | BS-PCR of *gapdh* |
| *gapdhs*-BSP-F | AAAATTTGGTGTAAGTATAAATTAATTGG |  |
| *gapdhs*-BSP-R | CAATAAAATTACATAATTAATCATAAAAAAAT |  |
| *ldha*-BSP-outer -F | GTGGGGGGTGTTAGGGTGAG | BS-PCR of *ldha* |
| *ldha*-BSP-F | TTTTTATAGATTGGAGTGAGATTATAGA |  |
| *ldha*-BSP-R | TCCTCTCTTAAATTCATATTTATAACTAAC |  |
| Q*stat1b*-F | CCTGTGGTCCGTTCAACAAAGC | QPCR of *stat1b* |
| Q*stat1b*-R | ATAGTAGCGACCAAAGGCGACG |  |
| Q*trap1*-F | GCAGAGGGTCGGGAAAAGAATA | QPCR of *trap1* |
| Q*trap1*-R | TGCTTGGAACTCGTGTTTGGAG |  |
| Q*gapdhs*-F | ATGTCTTCTCAGATTCAGGACCACC | QPCR of *gapdh* |
| Q*gapdhs*-R | ATGGTGAACTTCTCCCTTGTAACG |  |
| Q*ldha*-F | AATGTGGCTGGCGTGTCCCT | QPCR of *ldha* |
| Q*ldha*-R | CTTGACCAAAGTAGAAACTGGATGACA |  |
| Q*actin*-F | AGCCATCCTTCTTGGGTATG | QPCR of *actin* |
| Q*actin*-R | GGTGGGGCGATGATCTTGAT |  |
| q*NS1*-F | GTCACTGCCCATTGCTTCGC | QPCR of *NS1* |
| q*NS1*-R | GCGGCACGGGATCTGTTGTA |  |
| q*VP5*-F | CCCGGAACAAGGCTCACCAT | QPCR of *VP5* |
| q*VP5*-R | GCGTGAGCAGTCTCCAGCTT |  |
| 3.1-*gapdhs*-F | TTTAAACTTAAGCTTGGTACCATGTCTGAGCTTTGTGTTGGAATCA | Overexpression of *gapdhs* |
| 3.1-*gapdhs*-R | TGCTGGATATCTGCAGAATTCTTAAGCGTAATCTGGAACATCGTATGGGTACTCTTTGGAGTGCATGTAC |  |
| 3.1-*ldha*-F | TTTAAACTTAAGCTTGGTACCATGGCCTCTACAAAGGAGAAACTCA | Overexpression of *ldha* |
| 3.1-*ldha*-R | TGCTGGATATCTGCAGAATTCTCAAGCGTAATCTGGAACATCGTATGGGTACAGGGTCAGCTCCTTCTGAAC |  |
| si*gapdhs*-1-F | GCCUGCAGAAGGGAAUUAATT | siRNA1 of *gapdhs* |
| si*gapdhs*-1-R | CCCUCAAUGACAACUUUGUTT |  |
| si*gapdhs*-2-F | UUAAUUCCCUUCUGCAGGCTT | siRNA2 of *gapdhs* |
| si*gapdhs*-1-R | ACAAAGUUGUCAUUGAGGGTT |  |
| si*ldha*-1-F | CAAGGAUCUGACUGAUGAATT | siRNA1 of *ldha* |
| si*ldha*-1-R | UUCAUCAGUCAGAUCCUUGTT |  |
| si*ldha*-2-F | CAGUUGACAUCUUAACCUATT | siRNA2 of *ldha* |
| si*ldha*-1-R | UAGGUUAAGAUGUCAACUGTT |  |
